# Benchmarking the nutrition-related commitments and practices of major Belgian food companies

**DOI:** 10.1186/s12966-022-01269-1

**Published:** 2022-04-07

**Authors:** Iris Van Dam, Naomi Reimes, Stefanie Vandevijvere

**Affiliations:** 1 Sciensano, Service of Lifestyle and Chronic Diseases, Brussels, Belgium; 2 Université Paris-Saclay, INRAE, UR ALISS, 94205 Ivry-sur-Seine, France; 3 Rijksinstituut Voor Volksgezondheid en Milieu (RIVM), Bilthoven, Netherlands

**Keywords:** Business impact assessment, Food industry, Nutritional quality, Food supply, Nutrient profile, Accountability

## Abstract

**Background:**

To benchmark and quantitatively assess the transparency, specificity and comprehensiveness of nutrition-related commitments, as well as related practices of the largest Belgian food companies.

**Methods:**

The ‘Business Impact Assessment on Obesity and population-level nutrition’ (BIA-Obesity) was applied to evaluate nutrition-related commitments and practices concerning product formulation, labelling, promotion and accessibility by the biggest Belgian food and non-alcoholic beverage manufacturers (*n* = 19), supermarkets (*n* = 5) and quick-service restaurants (*n* = 7). Publicly available commitments were collected and company representatives given the opportunity to verify and complete the information (2019–2020). Commitments were scored according to the BIA-Obesity. To assess company practices, the following indicators were calculated: median Nutri-Score of product portfolios, the proportion of products not-permitted to be marketed to children (using the World Health Organisation Regional Office for Europe nutrient profile model), the proportion of ultra-processed food products (using the NOVA-classification) and the proportion of products displaying Nutri-Score on the front-of-pack. Promotions in supermarket flyers were analysed over a one-year period and quick-service restaurant density around schools was calculated. Correlations between commitments and performance indicators were calculated applying the Spearman's rank correlation coefficient.

**Results:**

Eighteen out of 31 companies participated (56%). Overall BIA-Obesity scores for commitments ranged from 2 to 75% (median = 35%) with notable variation across policy domains and food industries. The proportion of portfolios consisting of A and B Nutri-Score products ranged from 0 to 100% (median = 29%). The median proportion of products not-permitted to be marketed to children was 81% (range = 12%-100%) and the median proportion of ultra-processed foods was 75% (range = 2%-100%) across product portfolios. No significant correlations were observed between the strength of commitments and related performance indicators.

**Conclusion:**

Food industry actions do not meet recommended best practices. Performance indicators show large potential for improvement across policy domains and industries. Government regulations are urgently needed to improve food industry efforts and ensure that commitments translate into improved practices.

**Supplementary Information:**

The online version contains supplementary material available at 10.1186/s12966-022-01269-1.

## Introduction

In Belgium one in two adults and one in five youngsters (2–17 years of age) are overweight [1]. Both overweight and obesity significantly increase the risk of non-communicable diseases (NCDs) [2, 3]. This has indisputable economic consequences with a one unit Body Mass Index (BMI) reduction in Belgium being associated with a 15.9 billion euro total economic benefit over a time span of 20 years [4, 5]. It has been established that unhealthy food environments support the increase in overweight and NCDs as they make the unhealthy choices easier than the healthy choices [6]. Actions from the government, society and the food industry together with individual factors such as income, preferences and habits influence the healthiness of food environments [7]. A regulatory environment that supports profit growth enables the food industry to influence food environments without due consideration of the impact on health [7–9]. Many food companies have made commitments to improve some aspects of food environments through voluntary reformulation, labelling and marketing initiatives, but commitments are often non-specific and fall short of best-practice recommendations [10–12]. To ensure that commitments translate into real-world improvement of food environments it is essential to monitor and evaluate commitments made by food companies as well as related company practices and performances [10, 13].

In addition to commitments made by individual companies, a number of overarching industry pledges and voluntary public policy initiatives to improve food environments are in place in Belgium. These include the Nutri-Score [14, 15], the ‘Convention for a balanced diet’ [16] and the ‘Belgian Pledge’ [17]. The Nutri-Score classifies food and drink products in five categories based on the nutrient content per 100 g/ml and is the official front-of-pack labelling system in Belgium since 2019 [14, 15]. Categories are distinguished by five letters (colours) with ‘A’ (dark green) being the most healthy and ‘E’ (red) the least healthy category [15]. As part of the ‘Convention for a balanced diet’ the Ministry of Public Health encourages the food industry to commit to reformulate products within selected food categories and reduce portion sizes [16]. The ‘Belgian Pledge’ in turn is an industry initiative to limit marketing of products to children that do not meet the nutrition criteria in media where at least 35% of the audience is under 12-years of age [17]. The nutrition criteria enforced by the ‘Belgian Pledge’ are the same as the ‘EU Pledge’ which has been scrutinized for not effectively protecting children from unhealthy food marketing due to lenient nutrition criteria and the target audience definition [18, 19]. An alternative, more stringent nutrient profiling model that allows fewer products to be marketed to children, is the World Health Organisation Regional Office for Europe nutrient profile model (WHO-model) [19, 20].

To date the transparency, comprehensiveness and specificity of the nutrition-related commitments made by the Belgian food industry, both made by individual companies as through overarching industry pledges, have not yet been evaluated. Neither has it been assessed if stronger nutrition-related commitments translate into stronger practices and performance. This study set out to benchmark and quantitatively assess the nutrition-related commitments concerning product formulation, labelling, promotion and accessibility made by the biggest Belgian food and non-alcoholic beverage manufacturers, supermarkets and quick-service restaurants, as well as their practices within these same policy domains. To our knowledge this study is the first to make a combined assessment of both nutrition-related commitments and practices of the food industry.

## Methodology

To assess food industry commitments and practices the ‘Business Impact Assessment on Obesity and population-level nutrition’ (BIA-Obesity) was applied. The BIA-Obesity has been developed by the International Network for Food and Obesity/Non-communicable Diseases Research, Monitoring and Action Support (INFORMAS) and was previously described in detail by Sacks et al. [7, 10]. The tool consists of six domains across which commitments and practices are assessed. The ‘Corporate strategy’ domain considers companies’ overall nutrition strategy, taking into account specific targets and reporting practices. The ‘Product formulation’ domain assesses voluntary reformulation commitments related to sodium, saturated fat, trans-fat, added sugar and energy content. In case companies made commitments to reduce palm-oil within their product portfolio this was taken into account for the indicator regarding saturated fat reduction. The ‘Nutrition labelling’ domain evaluates the application of voluntary front-of-pack labelling systems, the extent to which the use of nutrition and health claims is linked to the healthiness of products, menu labelling practices (for quick-service restaurants) and the use of shelf labels (for supermarkets). The’Product and brand promotion’ domain considers commitments for reducing the exposure of children to unhealthy food marketing, including the in-store environment of supermarkets and quick-service restaurants. Within the ‘Product accessibility’ domain commitments regarding food pricing and availability of healthy versus less healthy foods are evaluated. The ‘Relationships with other organisations’ domain assesses the transparency regarding funding provided to external groups such as nutrition and physical activity programs, external research and industry groups [10].

All indicators relate to commitments that go beyond legislative requirements. Consequently, indicators and scoring criteria were adapted to the Belgian context. Indicators related to the on-pack disclosure of the ingredients list, trans-fat and added sugar content were removed as this is regulated by the European Union [21]. As it is not common practice in Belgium for supermarkets to have in-store restaurants, also indicators relating to menu-labelling of restaurant foods in supermarkets were removed. The remaining indicators were adapted to suit the Belgian regulatory environment and take into account relevant industry pledges (i.e. Belgian Pledge) and voluntary government-led initiatives (i.e. Nutri-Score, Convention for a Balanced Diet).

In addition to the commitments, dependent on Belgian data availability, a selection of performance indicators were calculated across BIA-Obesity domains. The healthiness of product portfolios was assessed within the domain ‘Product formulation’, the proportion of products not-permitted to be marketed to children and the promotions within supermarket flyers analysed within the domain’Product and brand promotion’, the proportion of products displaying Nutri-Score assessed within the domain ‘Product labelling’ and the quick-service restaurant density around schools evaluated within the domain ‘Product accessibility’.

This study was approved by the Human Ethics Committee of the University of Ghent (number: 2019/0780).

### Selection of food companies

Food companies with a combined market share of over 40% among packaged food manufacturers (44%) and non-alcoholic beverage manufacturers (50%), supermarkets (49%) and quick-service restaurants (52%) were selected using Belgian Euromonitor 2018 market share data (Table 1) [22]. For packaged food manufacturers, an additional selection was conducted based on companies’ market share within specific food categories to ensure that the most prominent companies per food category were covered by the selection (*‘Breakfast cereals’, ‘Baked goods’ ‘Confectionery’, ‘Ice-cream and frozen desserts’, ‘Processed Fruit and Vegetables’, ‘Processed Meat and Seafood’, ‘Sweet biscuits and cereal bars’, ‘Drinking milk products’, ‘Yoghurts’, ‘Savoury snacks’ and ‘Ready meals’*). Four additional companies were included based on this extra selection (Dr. Oetker, Bonduelle, Imperial Meat Products and McCain).

**Table 1:** The market shares per food industry as determined by Euromonitor and most sold product categories of companies included in the study (Belgium, Euromonitor, 2018)

Companies	Market share (%)	Most sold (own-brand) product categories
**Packaged food manufacturers**		
***Mondelēz***	3.1	Bread & bakery products, Confectionary, Dairy
***Unilever***	2.5	Dairy, Convenience foods, Sauces
***Nestlé***	2	Dairy, Non-alcoholic beverages, Cereal & grain products
***Danone***	1.9	Dairy
***Friesland Campina***	1.3	Dairy
***PepsiCo***^a^	1.1	Non-alcoholic beverages, Savoury snack foods
***Ter Beke***	1.1	Convenience foods
***Ferrero***	1	Bread & bakery products, Confectionary
***GB Foods**** (Previously Continental Foods)*	1	Sauces, Convenience foods
***Mars***	0.9	Confectionary, Sauces, Cereal & grain products
***Lotus Bakeries***	0.9	Bread & bakery products
***Kellogg’s***	0.8	Cereal & grain products
***Iglo***	0.7	Fruits & vegetables, Convenience foods, Meat & fish products
***Dr. Oetker***^b^	0.7	Convenience foods, Bread & bakery products, Dairy
***Bonduelle***^c^	0.3	Fruits & vegetables
***Imperial Meat Products***^d^	0.9	Meat & fish products
***McCain***^c^	0.2	Fruits & vegetables
***N* = *17***	***20.4***^e^	
**Non-alcoholic beverage manufacturers**		
***Coca-Cola***	35.4	Non-alcoholic beverages
***PepsiCo***^a^	3.2	Non-alcoholic beverages, Savoury snack foods
***Schweppes**** (Suntory Holdings)*	3	Non-alcoholic beverages
***N* = *3***	***41.6***^f^	
**Supermarkets**		
***Colruyt***	15.9	Fruits & vegetables, Non-alcoholic beverages, Dairy
***Delhaize***	15.6	Fruits & vegetables, Non-alcoholic beverages, Dairy
***Aldi***	6.3	Dairy, Non-alcoholic beverages, Fruits & vegetables
***Carrefour***	6.2	Fruits & vegetables, Non-alcoholic beverages, Convenience foods
***Lidl***	3.8	Dairy, Non-alcoholic beverages, Bread & bakery products
***N* = *5***	***52.4***	
**Quick-service restaurants**		
***McDonald's***	17	Burgers
***Quick***	12	Burgers
***Panos***	9	Bread & bakery products
***Pizza Hut***	6	Pizza
***Exki***	6	Bread & bakery products, Convenience foods
***Domino's Pizza***	17	Pizza
***Paul***	12	Bread & bakery products
***N* = *7***	***49.4***	

^a^PepsiCo was scored as both food and a non-alcoholic beverage manufacturer

^b^The largest market share within the Euromonitor food category ‘Ready meals’

^c^Having among the largest market share within the Euromonitor food category ‘Processed Fruit and Vegetables’

^d^Having among the largest market share within the Euromonitor food category ‘Processed Meat and Seafood’

^e^and ^f^Excluding the supermarkets as food and beverage manufacturers (market share foods: 23.3%; market share beverages: 8.8%)

### Data collection and analyses

#### Nutrition-related commitments

Publicly available commitments and policies were collected between March 2019 and October 2020. Company websites (national and global), brand websites, financial and corporate social responsibility reports, industry association websites and media articles were taken into account as well as abovementioned industry pledges and initiatives.

All relevant information was saved by downloading documents and through screenshots of the webpages. Commitments were entered in an Excel spreadsheet per BIA-Obesity indicator. A report was written for each company summarizing the collected information per BIA-Obesity domain and providing an overview of the scoring. Company representatives were contacted via various channels, including meetings with industry associations (Bemora, Comeos, Fevia), phone call inquiries, contact information on company/brand websites and LinkedIn. Emails were sent to representatives explaining the study. Companies willing to participate signed a written informed consent and were sent the summary report and complete Excel file providing them with the opportunity to verify and complete the collected data. All additional information had to be substantiated with supporting documents. When requested by company representatives, non-disclosure agreements could be signed for sensitive information that was provided to improve the BIA-Obesity scoring. For companies that refused participation or failed to share feedback in time, the assessment was based solely on publicly available information. Supermarkets were assessed as both retailers and food manufacturers (own-brand products).

The nutrition-related commitments were scored in Excel. Supplementary file 1 provides examples of how scores were assigned. All company commitments were scored independently by IVD and NR. Discrepancies were discussed till an agreement was obtained. The final BIA-Obesity scores per domain were weighted as recommended by INFORMAS (Supplementary file 2).

Median scores (range), overall and per BIA-Obesity domain, were calculated including all food industries and separately for food and non-alcoholic beverage manufacturers, supermarkets and quick service restaurants. For companies that verified and completed the public information, median scores before and after participation were calculated. A one-tailed Wilcoxon signed-rank test was conducted to compare scores before and after participation. A two-tailed Wilcoxon rank-sum test was used to compare scores of companies that engaged with the process to scores of those that did not engage.

#### Practices

Practices were assed across the BIA-Obesity domains ‘Product formulation’ and’Product and brand promotion’ for all food industries. To some extent practices were assessed within the domain ‘Product labelling’ for food and beverage manufacturers and ‘Product accessibility’ for quick-service restaurants. No performance indicators were included for the domains ‘Corporate strategy’ and ‘Relationships with other organisations’ due to feasibility and data availability. An overview of all performance indicators can be found in Table 2.

**Table 2:** An overview of the performance indicators per food industry and ‘Business Impact assessment on Obesity and Population Nutrition’ (BIA-Obesity) domain. The data source and the year of data collection are specified per indicator

Food Industry	BIA-Obesity Domain	Performance indicators	Data sources	Years
**Food and beverage manufacturers**	**Product formulation**	*For full product portfolio:* ✓ Median Nutri-Score ✓ % of products with Nutri-Score A and B ✓ % of products with Nutri-Score D and E ✓ % of products that are ultra-processed	Nutritrack branded food composition database Belgium	2018
	**Nutrition labelling**	*For full product portfolio:* ✓ % of products with Nutri-Score displayed on the front-of-pack	Pictures of all food products with Nutri-Score on the front-of-pack in-store	2019
	**Product and brand promotion**	*For full product portfolio:* ✓ % of products not-permitted to be marketed to children according to the World Health Organisation Regional Office for Europe nutrient profile model (WHO-Model)	Nutritrack branded food composition database Belgium	2018
**Supermarkets**	**Product formulation**	*For full own-brand product portfolio:* ✓ Median Nutri-Score ✓ % of Nutri-Score A and B ✓ % of Nutri-Score D and E ✓ % of products that are ultra-processed	Nutritrack branded food composition database Belgium	2018
	**Nutrition labelling**	*For full own-brand product portfolio:* ✓ % of products with Nutri-Score displayed on the front-of-pack	Pictures of all food products with Nutri-Score on front-of-pack in-store	2019
	**Product and brand promotion**	*For full own-brand product portfolio:* ✓ % of products not permitted to be marketed to children according to the WHO-Model	Nutritrack branded food composition database Belgium	2018
		*For all food products:* ✓ % of promotions for foods that are ultra-processed ✓ % of promotions for fresh fruit and vegetables ✓ % of promotions with promotional characters	Supermarket circulars	2019–2020
**Quick service restaurants**	**Product formulation**	*For meals and food portfolio online:* ✓ Median Nutri-Score ✓ % of meals with Nutri-Score A and B ✓ % of meals with Nutri-Score D and E	Company websites	2020^a^
	**Product and brand promotion**	*For meals and food portfolio online:* ✓ % of foods and meals not-permitted to be marketed to children according to the WHO-Model	Company websites	2020
	**Product accessibility**	*Outlet density around schools:* ✓ Proportion of outlets within 500 m road network distance from primary schools (Flanders only) ✓ Proportion of outlets within 500 m road network distance from secondary schools (Flanders only)	Locatus food retail database	2020

^a^2017 for Quick. No data available for Exki and Pizza Hut

##### Product formulation

Portfolios of food and beverage manufacturers, including supermarkets, were analysed using the Belgian Nutritrack branded food database 2018. This database contains products from the five biggest retailers in Belgium. It was compiled using pictures taken of all food products available in Carrefour, Lidl and Aldi. For Delhaize the nutritional data on own-brand products were received from the retailer. For Colruyt web scraping of nutritional information and ingredient lists from the online grocery store was applied. For the company Ter Beke only two food products were present in the Nutritrack branded food database 2018 as many of their product are sold outside of supermarkets. Consequently, this company was not discussed within the performance results, but data were included in graphs and tables. Alcoholic beverages, infant formula and baby foods were excluded.

For quick-service restaurants a database was compiled in 2020 using the nutritional information available on the national websites (Domino's Pizza, McDonald’s and Panos). The nutritional information on the national website of Paul was incomplete. The missing nutritional information was completed using data from the French website. On the website of Quick no nutritional information was available per 100 g and no portion sizes were specified. Instead, an online table with nutritional information for Belgium and Luxembourg from 2017 was used [23]. For Exki and Pizza Hut no nutritional information was available per 100 g, no portion sizes were specified and no other data could be obtained.

The Nutri-Score [14, 15], the WHO-model [20] and the NOVA-classification [24] were applied to assess the nutritional quality of company portfolio’s. The NOVA-classification distinguishes products based on their level of processing (unprocessed or minimally processed foods, processed culinary ingredients, processed foods and ultra-processed foods) [24]. The proportion of portfolios that are ultra-processed (NOVA), the proportion of products not-permitted to be marketed to children (WHO-Model), as well as the median Nutri-Score and the proportion of products with Nutri-Score A and B and D and E, were examined by company.

##### Product labelling

In November and December 2019, pictures were taken of all products carrying the Nutri-Score on the front-of-pack in stores of the five biggest supermarkets (Delhaize, Carrefour, Colruyt, Aldi, Lidl). Data were entered into a database and the distribution of the Nutri-Score was assessed.

##### Product and brand promotion

Food promotions in weekly or two-weekly supermarket flyers available from supermarket websites were collected over a one-year period (2019–2020) for the five biggest supermarkets. All promotions were entered into a database and classified according to the NOVA-classification and the 17 food categories of the WHO-model (Supplementary file 3). Per product the following information was recorded: product- and brand name, food category, Nutri-Score, the type of promotional character and the type of premium offer [25]. The proportion of promotions for ultra-processed foods, foods per WHO-model product category and for fresh fruits and vegetables as well as the proportion of promotions with promotional characters and premium offers were calculated. Data were analysed separately per supermarket and a distinction was made between promotions on the cover and on the inside of flyers. Methods were previously detailed by Vandevijvere et al. [26].

##### Product accessibility

The accessibility to quick-service restaurants near schools was assessed through the proportion of all company outlets within 500 m road network distance from the entrance of primary and secondary schools in Flanders. For this the Locatus database of food retailers for Flanders (2020) was used [27]. Locatus collects information on different types of retail outlets for commercial purposes across Flanders. It includes information on location, type, size, and opening times of all retailers through systemic area scans, which are conducted by employees of Locatus via field audits. Food outlets in shopping areas are audited every year, while other food outlets are audited every two to three years. The company Paul was excluded for this analysis as there were only two outlets identified in Flanders.

#### The relationship between commitments and practices

Correlations between commitments and practices were calculated applying the Spearman's rank correlation coefficient. Correlations were calculated between commitments made within the domain ‘Product formulation’ and the proportion of products within the portfolio with Nutri-Score A and B and D and E as well as the median Nutri-Score of the portfolio. Correlations between this domain and the proportion of ultra-processed products were also calculated. Lastly, correlations between commitments within the domain ‘Product and brand promotion’ and the proportion of products not-permitted to be marketed to children (WHO-model) were assessed.

*R*-values > 0.5 were considered to represent a strong correlation. *P*-values < 0.05 were considered statistically significant. All analyses were performed using SAS 9.4.

## Results

### Nutrition-related commitments

**Fig. 1:**
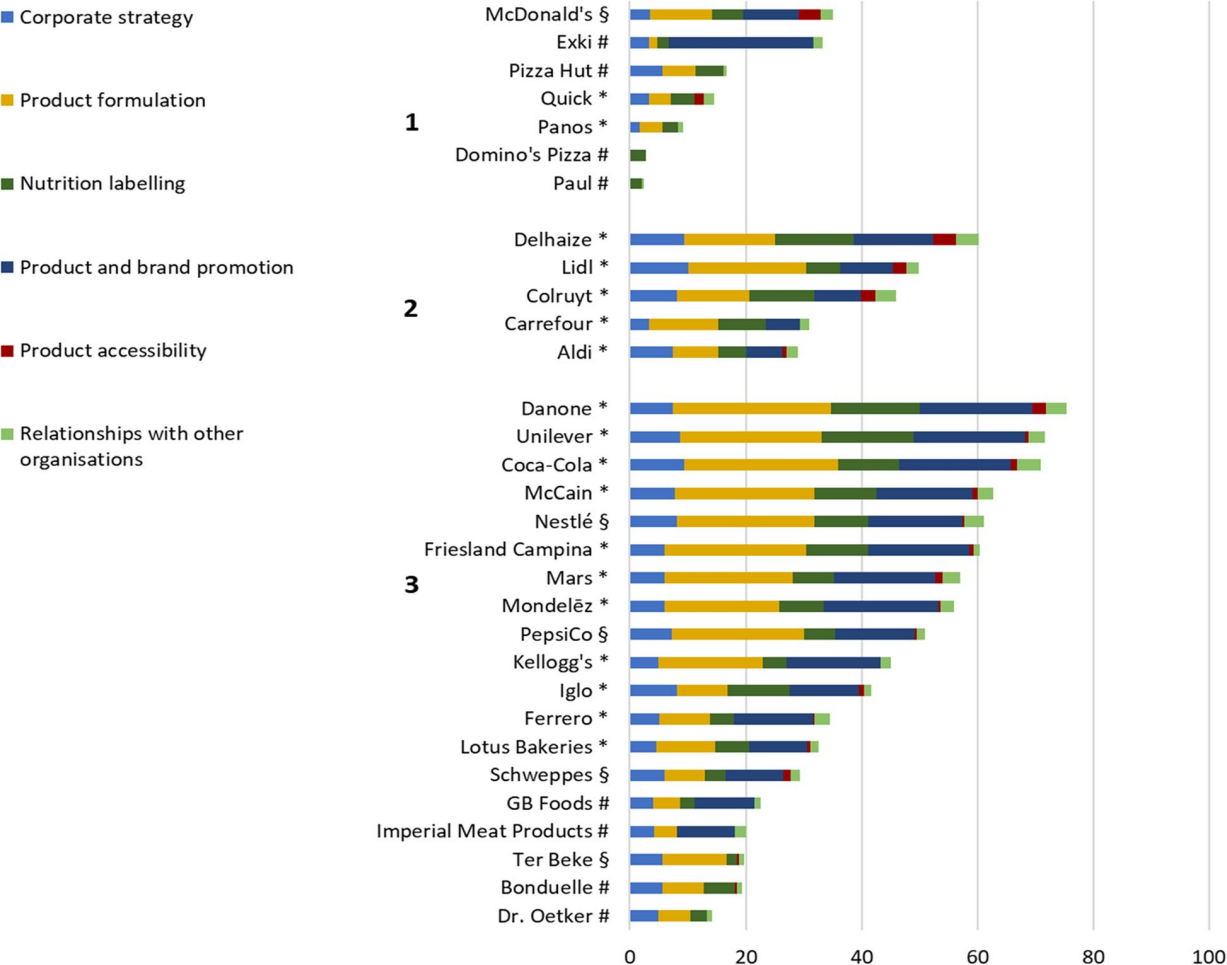
Overall and domain-specific ‘Business Impact assessment on Obesity and Population Nutrition’ (BIA-Obesity) scores for 1. Quick service restaurants, 2. Supermarkets, 3. Food and beverage manufacturers. * Full engagement with the process (*N* = 18); # Declined participation (*N* = 8); § Accepted participation, but contributions not received in time (*N* = 5); For # and §: Assessment of commitments was based on publically available information only

Out of the 31 selected companies, 18 verified and completed the publicly available information, five accepted participation but did not provide feedback in time and eight declined participation (Fig. 1).

Overall BIA-Obesity scores ranged from 2% to 75% (median = 35%). The median overall score was 45% (range = 14–75%) for food and beverage manufacturers, 46% (range = 29–60%) for supermarkets and 15% (range = 2–35%) for quick service restaurants (Fig. 1). Scores per BIA-Obesity domain and per company are presented in Table 3. For the 18 companies that participated (response rate = 56%), the median overall BIA-Obesity score significantly increased from 34% (scoring based on public information) to 51% (*p* < 0.001). Overall BIA-Obesity scores were significantly higher for companies that participated compared to companies that did not (*p* < 0.05) (data not shown).

**Table 3:**
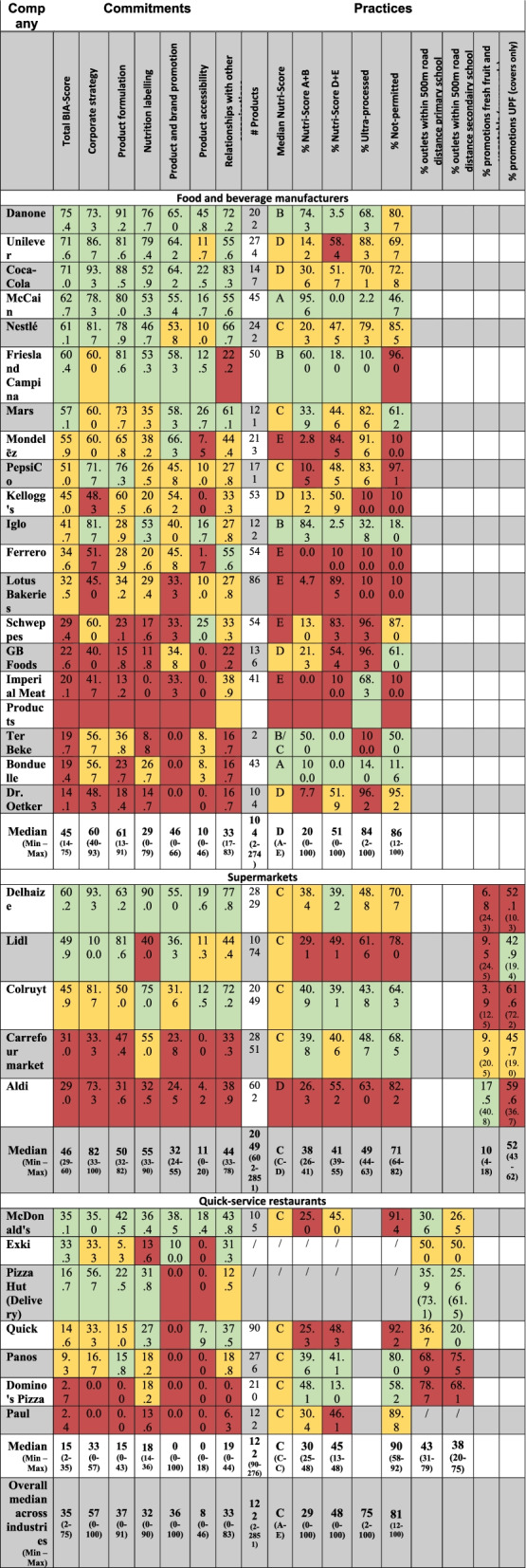
An overview of the ‘Business Impact assessment on Obesity and Population Nutrition’ (BIA-Obesity) scores for commitments and practices per company

Data are sorted by descending total BIA-Obesity score per food industry (food and beverage manufacturers, supermarkets and quick-service restaurants). Green indicates a score within the top third of companies per food industry and red indicates a score within the lowest third of companies per food industry. Yellow indicates the companies in between

The domain ‘Corporate strategy’ (median = 57%, range = 0–100%) was the best performing BIA-Obesity domain and the domain ‘Product accessibility’ (median = 8%, range = 0–46%) the worst. All companies, apart from two, made commitments within the ‘Corporate strategy’ domain. Supermarkets (median = 82%) performed better than food and beverage manufacturers (median = 60%) and quick-service restaurants (median = 33%) within this domain. Best performing companies recognised both national (‘Convention for a balanced diet’ or ‘Nutri-Score’) and international (‘The United Nations Sustainable Development Goals’ or ‘the WHO global NCD action plan’) priorities while regularly reporting on the progress toward their nutrition-related targets. The lowest performing companies made little or no mention of nutrition-related issues.

Within the ‘Product accessibility’ domain only limited commitments were in place. Supermarkets had the highest median score (11%), closely followed by food and beverage manufacturers (median = 10%). Quick-service restaurants scored the lowest (median = 0%). Ten out of 31 companies had no commitments within this domain. Among supermarkets, one committed to have checkouts free from unhealthy items while another committed to link in-store price promotion and promotions through loyalty program to the healthiness of products as determined by the Nutri-Score. Among the quick-service restaurants, one restaurant committed to not provide free refills. The implementation of taxes on some unhealthy food products was supported by two companies and opposed by seven.

The median score within the domain ‘Product formulation’ was 37% (0–91%) with food and beverage manufacturers scoring the highest (61%) followed by supermarkets (50%) and quick-service restaurants (15%). Two quick-service restaurants did not have any commitments in this area. Seven out of the 19 food and beverage manufacturers, four out of five supermarkets and one out of seven quick-service restaurants made commitments to reduce all applicable nutrients of concern (sodium, saturated fat, trans fat, added sugar and energy content). Commitments to reduce the energy content and portion size where in place least of all. Two out of the 19 food and beverage manufacturers and two out of five supermarkets already applied the Nutri-Score to guide reformulation.

The domain ‘Nutrition labelling’ obtained a median score of 32% (range = 0–90%). Only one company had no commitments within this area. When comparing food industries it was clear that supermarkets performed better (median = 55%) than food and beverage manufacturers (median = 29%) and quick-service restaurants (median = 18%). The highest score was obtained by a supermarket (90%) that committed to apply the Nutri-Score on own-brand food products as well as to all available products using in-store shelf tags and the company website. All supermarkets and six out of the 19 food and beverage manufacturers committed to the implementation of the Nutri-Score. None of the quick-service restaurants committed to menu labelling, but all provided online nutritional information to some extent. One company publicly committed not to display nutrition and health claims on products defined as unhealthy according to their own classification system.

The domain ‘Product and brand promotion’ obtained a median score of 36% (range = 0–100%). Food and beverage manufacturers obtained a median score of 46%, supermarkets of 32% and quick-service restaurants 0%. Eight companies made no commitments to reduce marketing towards children (five quick-service restaurants and three food and beverage manufacturers). All supermarkets and almost all food and beverage manufacturers (15/19) were a signatory to the Belgian Pledge.

Only one quick-service restaurant was a signatory. One quick-service restaurant in turn specifically committed to not advertise at all. None of the selected companies developed marketing policies for children up to the age of 18 years.

Lastly, the median score for the domain ‘Relationships with other organisations’ was 33% (range = 0–83%). One quick-service restaurant had no publicly available information for this domain. Median scores per food industry ranged from 33% for food and beverage manufacturers up to 44% for supermarkets and down to 19% for quick-service restaurants. Few companies specifically committed to not making any political donations.

### Practices

The performance results per indicator and per company are presented in Table 3.

#### Product formulation

Across all food industries, the proportion portfolios consisting of A and B Nutri-Score products ranged from two companies with 0% of their portfolio having a score A or B up to one company having 100% products with Nutri-Score A or B (median = 29%).The median Nutri-Score of food and beverage portfolios ranged from A to E. All selected supermarkets and quick-service restaurants had a median Nutri-Score C apart from one supermarket with a median Nutri-Score D. The proportion of portfolios with Nutri-Score A and B ranged from 0% to 100% for food and beverage manufacturers (median = 20%), 26% to 41% for supermarkets (median = 38%) and from 25% to 48% for quick-service restaurants (median = 30%). According to the NOVA-classification, median portfolios of selected food and beverage manufacturers consisted for 84% of ultra-processed foods (one company with 2% up to four companies with 100% ultra-processed products). For supermarkets this was 49% (44%-63%). Median portfolios across all industries consisted for 75% of ultra-processed foods.

#### Product labelling

A total of 1781 products in the supermarkets displayed the Nutri-Score by the end of 2019. This represented about 10% of all products available on the Belgian market. About 90% of products displaying the Nutri-Score on pack were supermarket own-brand products. The two best performing food and beverage manufacturers had 34% of their products labelled with the Nutri-Score, for the best performing supermarket this was 30% of their portfolio. From the products displaying the Nutri-Score, 56% displayed Nutri-Score A or B. 26% displayed Nutri-Score D or E (data no shown).

#### Product and brand promotion

All food and beverage portfolios of companies not mainly selling fruits and vegetables consisted of at least 61% products not-permitted to be marketed to children. Food and beverage portfolios were for 86% (median) not-permitted to be marketed to children (range = 12%-100%). For supermarkets this median decreased to 71% (range = 64%-82%) not-permitted products. Quick-service restaurants had the highest proportion of products not-permitted to be marketed to children (median = 90%, range = 58%-92%). Overall 81% of portfolios were not suitable to be marketed to children.

Looking at food promotions in supermarket flyers over a one-year period, a total of 15.271 food promotions were analysed. According to the WHO-model, ‘Processed meat, poultry and fish’ (11.8%), ‘Fresh and frozen fruit and vegetables and legumes’ (9.5%) and ‘Soft drinks and sweetened beverages’ (9.0%) were promoted most regularly (data not shown). About 52% (range = 43%-63%) of all promotions were for ultra-processed products. Less than 10% of the promotions were for fresh fruits and vegetables (range = 4%-18%). Premium offers were used in 20% (range = 2%-42%) of the promotions and promotional characters in 5% (range = 1%-9%). The Nutri-Score was only visible for less than 2% of the promotions (data not shown). Products promoted on the cover of the flyers tended to be healthier than the promotions throughout the entire flyers. Data are presented in Table 3. Data were previously published and described in detail by Vandevijvere et al. [26].

#### Product accessibility

Among quick-service restaurants, four out of six companies had more than 50% of their outlets in Flanders located within 500 m road distance of primary and secondary schools (Table 3). Around both primary and secondary schools this percentage increased since 2008 for three quick-service restaurants.

### The relationship between commitments and practices

No significant correlations were observed between commitments within the domains ‘Product formulation’ and ‘Product and brand promotion’ and respective performance indicators. As five out of seven selected quick-service restaurants made no commitments within the domain ‘Product and brand promotion’, no correlations could be calculated with practices as determined by the WHO-model.

It can be observed from Table 3 that companies within the top third for commitments within the domain of ‘Product formulation’ don’t necessarily have the healthiest portfolios as determined by the Nutri-Score and NOVA-classification. On the contrary, there are companies within the lowest third for commitments that still have among the heathiest portfolios. The same can be observed for commitments and practices within the domain ‘Product and brand promotion’.

These results suggest that companies with more specific, transparent and comprehensive commitments to reformulate products and limit marketing towards children don’t necessarily have healthier portfolios with less ultra-processed products and a larger proportion of products permitted to be marketed to children.

## Discussion

This study was the first to quantitatively assess both nutrition-related commitments and practices of the largest Belgian food and beverage manufacturers, supermarkets and quick-service restaurants. A large variation was observed between companies according to the BIA-Obesity scores and performance indicators. Overall BIA-Obesity scores ranged from 2% to 75% (median = 35%). The domain ‘Corporate strategy’ performed best while the domain ‘Product accessibility’ performed worst. The performance indicators indicated unhealthy food environments with the majority of portfolios consisting of ultra-processed foods and products not-permitted to be marketed to children, only limited promotion of fresh fruits and vegetables in supermarket flyers and several quick-service restaurants having most of their outlets within 500 m road distance of schools.

Median overall BIA-Obesity scores as well as the scores per domain in Belgium were similar to results previously found in Australia and New Zealand and higher than the scores found in Malaysia [11, 12, 28]. Similar to these previous studies, this study showed that BIA-Obesity scores significantly increased for companies that engaged with the process and verified and completed the publicly available data [12, 28]. The response rates were slightly higher in Belgium (56%) compared to Australia (47%) and New Zealand (48%) and significantly higher than in Malaysia (18%) [11, 12, 28] (Supplementary file 4). Nonetheless, across all four countries the domain ‘Corporate strategy’ was identified as the best performing domain and ‘Product accessibility’ as the worst. These findings are in line with the results of the global Access To Nutrition Index (ATNI) which in 2018 and 2021 identified the domain ‘Governance’ as the highest scoring category and ‘Accessibility’ the lowest [29, 30]. In a similar manner to the BIA-Obesity, the ATNI benchmarks food company commitments and practices, but does this at global level for only food and beverage manufacturers while looking at both over- and undernutrition [13, 31].

The ATNI in 2018 also applied the WHO-model to assess practices and found that globally the portfolios of selected companies consisted for more than 50% of products not-permitted to be marketed to children [29]. These findings are similar to our results that found that food and beverage portfolios of companies not mainly selling fruits and vegetables consisted of at least 61% products not-permitted to be marketed to children. Previous research also found that on average 36% of foods consumed in Belgium in 2014–2015 were ultra-processed according to the NOVA-classification and contributed to 30% of the daily energy intake [32]. These results are not surprising as our data showed that median portfolios of the biggest Belgian food and beverage manufacturers (including supermarkets) consisted for 75% of ultra-processed foods. This is however of concern with an increasing number of studies showing an association between consumption of ultra-processed foods and overweight [33–35].

Companies should strengthen their role in improving food environments by enhancing their nutrition-related commitments. Current voluntary commitments fall short of recommended best practices. It is recommended for all companies to commit to SMART (Specific, Measurable, Achievable, Relevant and Time bound) reformulation targets to reduce nutrients of concern (salt, sugar, trans fat, saturated fat and energy) using an official nutrient profiling system (such as the Nutri-Score), develop a marketing policy using the WHO-model that applies to all children below the age of 18 and support evidence-based fiscal policies. For food and beverage manufacturers it is recommended to only label products with nutrition and health claims when products are healthy.

Specifically for quick-service restaurants it is recommended to disclose nutritional information on menus and commit to not open new outlets in the vicinity of schools. Lastly, for supermarkets, it is recommended to dedicate a minimum amount of floor space to healthy products and limit the placement of unhealthy products at high-traffic areas such as end of aisles and cash registers. It is expected that strengthened commitments will translate into improved practices and performance. Nevertheless, this needs to be closely monitored as research in the UK has shown that voluntary reformulation policies between 2015 and 2018 did not translate into noteworthy changes in the nutritional quality of products sold by the top ten food and beverage manufacturers [36]. Also in Canada it was found that companies with stronger commitments within the area of product reformulation did not have portfolios with a better nutritional quality [37]. Moreover, research has pointed out the importance of being cautious with voluntary company commitments or commitments made through public–private partnerships. Such commitments can legitimise a company's role in the formulation of public health policies as well as provide them with an official platform to advertise their efforts to improve health and wellbeing, irrespective of the ongoing efforts going beyond business as usual and truly having an impact on health [38, 39]. Furthermore, it has been suggested that voluntary commitments, instead of strengthening public health, have rather undermined policy implementation in areas most effective to improve population health, such as marketing restrictions and fiscal policies to make unhealthy foods relatively more expensive compared to healthier alternatives [39, 40]. As stated by Douglas et al., “the industry is not a disinterested partner in public health” [38]. As such it is a misconception that the food industry will place population health above its own interests [40]. Regardless of the opposition, government regulation can provide the necessary tools to motivate the food industry to strengthen actions and ensure that commitments move towards recommended best practices [41].

This study has several strengths and weaknesses. A key strength is that it is the first study to take into account a wide-range of performance indicators in addition to the nutrition-related commitments across the BIA-Obesity domains. It is anticipated that, because of liaising with company representatives, there is a higher chance of the recommendations to be implemented at company-level. Nonetheless, also important limitations were identified. Only about half of the selected companies verified and completed the publicly available data, resulting in the assessment of the remaining companies being based on publicly available information only. In addition, the assessment of practices was a snapshot in time and does not capture potential changes over time due to strengthened company policies. It is recommended for future applications of the BIA-Obesity to consider changes overtime in these performance indicators and to assess associations between these changes and the commitments made across BIA-Obesity domains. This iteration was not able to capture practices related to corporate political activities (such as lobbying, political donations and funding of research) that may influence food policies. Towards the future it is recommended to include such performance indicators linking to the BIA-Obesity domain of ‘Relationships with other organisations’.

In conclusion, Belgium is currently relying on voluntary actions by the food industry to improve food environments. Voluntary actions that fall short of recommended best practices while performance indicators show there is still large potential for improvement. No associations were observed between the strength of nutrition-related commitments and practices. So, even though food companies may recognise their role in improving food environments, government regulation is urgently needed to improve their efforts and ensure that commitments translate into improved practices and performance and eventually food environments that one day might make healthy food choices easier than unhealthy ones.

## Supplementary Information

**Additional file 1:** **Supplementary file 1. **Examples of how publicly available commitments were collected and scored according to the Business Impact Assessment on Obesity and Population Level Nutrition (BIA-Obesity) tool (Belgium, 2020).

**Additional file 2:** **Supplementary file 2.** Weighting per ‘Business Impact Assessment on Obesity and Population Nutrition’ (BIA-Obesity) domain and food industry (Belgium, 2020).

**Additional file 3:** **Supplementary file 3.** The 17 food categories included in the World Health Organisation Regional Office for Europe nutrient profile model (WHO-model) [20].

**Additional file 4:** **Supplementary file 4.** Overall median ‘Business Impact assessment on Obesity and Population Nutrition’ (BIA-Obesity) scores across countries where data were collected for food and beverage manufacturers, supermarkets and quick-service restaurants and companies had the opportunity to verify and complete the publicly available data [11, 12, 28].

## Data Availability

The datasets used and/or analysed during the current study are available from the corresponding author on reasonable request.

## Abbreviations

ATNI: Access To Nutrition Index
BIA-Obesity: Business Impact Assessment on Obesity and population-level nutrition
BMI: Body Mass Index
INFORMAS: International Network for Food and Obesity/Non-communicable Disease Research, Monitoring and Action Support
NCDs: Non-Communicable Diseases
SMART: Specific, Measurable, Achievable, Relevant and Time bound
WHO-model: World Health Organisation Regional Office for Europe nutrient profile model

## References

[1] S. Drieskens, L. Gisle, R. Charafeddine, S. Demarest, E. Braekman, D. Nguyen, J. Van der Heyden, F., Berete, L. Hermans, J. Tafforeau., J. Van der Heyden, D. Nguyen, F. Renard, A. Scohy, S. Demarest, S. Drieskens, L. Gisle. Gezondheidsenquête 2018: Levensstijl. Samenvatting van de resultaten. Brussel, België: Sciensano; Report No.: D/2019/14.440/52. Available from: www.gezondheidsenquete.be

[2] World Health Organization. Non communicable diseases. 2021 [cited 23 Jul 2021]. Available from: https://www.who.int/news-room/fact-sheets/detail/noncommunicable-diseases

[3] Nyberg ST, Batty GD, Pentti J, Virtanen M, Alfredsson L, Fransson EI (2018). Obesity and loss of disease-free years owing to major non-communicable diseases: a multicohort study. Lancet Public Health.

[4] Verhaeghe N, De Greve O, Annemans L (2016). The potential health and economic effect of a body mass index decrease in the overweight and obese population in Belgium. Public Health.

[5] Branca F, Nikogosian H, Lobstein T, World Health Organization, editors. The challenge of obesity in the WHO European Region and the strategies for response. Copenhagen: WHO Regional Office for Europe; 2007. 323.

[6] Swinburn B, Egger G (2004). The runaway weight gain train: too many accelerators, not enough brakes. BMJ.

[7] Swinburn B, Sacks G, Vandevijvere S, Kumanyika S, Lobstein T, Neal B (2013). INFORMAS (International Network for Food and Obesity/non-communicable diseases Research, Monitoring and Action Support): overview and key principles: INFORMAS overview. Obes Rev.

[8] White M, Aguirre E, Finegood DT, Holmes C, Sacks G, Smith R (2020). What role should the commercial food system play in promoting health through better diet?. BMJ.

[9] Stuckler D, Nestle M (2012). Big food, food systems, and global health. PLoS Med.

[10] Sacks G, Vanderlee L, Robinson E, Vandevijvere S, Cameron AJ, Ni Mhurchu C (2019). BIA-Obesity (Business Impact Assessment—Obesity and population-level nutrition): a tool and process to assess food company policies and commitments related to obesity prevention and population nutrition at the national level. Obes Rev.

[11] Ng S, Sacks G, Kelly B, Yeatman H, Robinson E, Swinburn B (2020). Benchmarking the transparency, comprehensiveness and specificity of population nutrition commitments of major food companies in Malaysia. Glob Health.

[12] Sacks G, Robinson E, Cameron AJ, Vanderlee L, Vandevijvere S, Swinburn B (2020). Benchmarking the nutrition-related policies and commitments of major food companies in Australia, 2018. Int J Environ Res Public Health.

[13] Sacks G, Swinburn B, Kraak V, Downs S, Walker C, Barquera S (2013). A proposed approach to monitor private-sector policies and practices related to food environments, obesity and non-communicable disease prevention. Obes Rev.

[14] Federale Overheidsdienst Volksgezondheid, Veiligheid van de Voedselketen en Leefmilieu. Koninklijk Besluit Betreffende Het Gebruik van Het Logo “Nutri-Score”. 2019. Available from: https://www.etaamb.be/nl/koninklijk-besluit-van-01-maart-2019_n2019040711.html

[15] Federale overheidsdienst volksgezondheid, veiligheid van de voedselketen en leefmilieu. Nutri-Score frequently asked questions, Scientific and Technical. 2019. Available from: https://www.health.belgium.be/nl/node/36098

[16] Convenant Evenwichtige Voeding. Convenant Evenwichtige Voeding. [cited 8 Jul 2021]. Available from: https://www.convenantevenwichtigevoeding.be/nl

[17] Belgian Pledge. Belgian Pledge. [cited 8 Jul 2021]. Available from: https://www.belgianpledge.be/nl

[18] Landwehr SC, Hartmann M (2020). Industry self-regulation of food advertisement to children: Compliance versus effectiveness of the EU Pledge. Food Policy.

[19] European Commission. Joint Research Centre. Comparison of the nutrient profiling schemes of the EU Pledge and the World Health Organization regional office for Europe: a toolkit. LU: Publications Office; 2015 [cited 10 Feb 2021]. Available from: 10.2787/24358

[20] World Health Organization (2015). WHO Regional Office for Europe nutrient profile model.

[21] Regulation (EU) No 1169/2011 of the European Parliament and of the Council of 25 October 2011 on the provision of food information to consumers, amending Regulations (EC) No 1924/2006 and (EC) No 1925/2006 of the European Parliament and of the Council, and repealing Commission Directive 87/250/EEC, Council Directive 90/496/EEC, Commission Directive 1999/10/EC, Directive 2000/13/EC of the European Parliament and of the Council, Commission Directives 2002/67/EC and 2008/5/EC and Commission Regulation (EC) No 608/2004 Text with EEA relevance. OJ L 304. 2011. p. 18–63. https://eur-lex.europa.eu/eli/reg/2011/1169/oj.

[22] Euromonitor International. Passport Global Market Information Database. 2017. Available from: http://www.portal.euromonitor.com

[23] Quick. Tableau des valeurs nutritionnelles/Overzicht voedingswaarden 03–09–2017 au/tot 13–11–2017. 2017 [cited 16 Apr 2020]. Available from: https://www.quick.lu/docs/nutritionalvalues.pdf?1

[24] Monteiro CA, Cannon G, Levy R, Moubarac J-C, Jaime P, Martins AP, NOVA (2016). The star shines bright. World Nutr.

[25] Kelly B, Vandevijvere S, Ng S, Adams J, Allemandi L, Bahena-Espina L (2019). Global benchmarking of children’s exposure to television advertising of unhealthy foods and beverages across 22 countries. Obes Rev.

[26] Vandevijvere S, Van Dam I (2021). The nature of food promotions over one year in circulars from leading Belgian supermarket chains. Arch Public Health.

[27] Locatus. Locatus. [cited 23 Aug 2021]. Available from: https://locatus.com/

[28] Kasture A, Vandevijvere S, Robinson E, Sacks G, Swinburn B (2019). Benchmarking the commitments related to population nutrition and obesity prevention of major food companies in New Zealand. Int J Public Health.

[29] Access to Nutrition Foundation (ATNF). Access to Nutrition Index – Global Index 2018. Utrecht, The Netherlands: ATNF; 2018. Available from: https://accesstonutrition.org/app/uploads/2020/02/GI_Global-Index_Full_Report_2018.pdf

[30] Access to Nutrition Foundation (ATNF). Access to Nutrition Index – Global Index 2021. Utrecht, The Netherlands: ATNF; 2021. Available from: https://accesstonutrition.org/app/uploads/2021/06/Global-Index-2021-Executive-Summary.pdf

[31] Access to Nutrition Foundation. Access to Nutrition Initiative. Access to Nutrition. [cited 26 Feb 2021]. Available from: https://accesstonutrition.org/

[32] Vandevijvere S, De Ridder K, Fiolet T, Bel S, Tafforeau J (2019). Consumption of ultra-processed food products and diet quality among children, adolescents and adults in Belgium. Eur J Nutr.

[33] Hall KD, Ayuketah A, Brychta R, Cai H, Cassimatis T, Chen KY (2019). Ultra-processed diets cause excess calorie intake and weight gain: an inpatient randomized controlled trial of ad libitum food intake. Cell Metab.

[34] Monteiro CA, Moubarac J-C, Levy RB, Canella DS, da Costa Louzada ML, Cannon G (2018). Household availability of ultra-processed foods and obesity in nineteen European countries. Public Health Nutr.

[35] Vandevijvere S, Jaacks LM, Monteiro CA, Moubarac J, Girling-Butcher M, Lee AC (2019). Global trends in ultraprocessed food and drink product sales and their association with adult body mass index trajectories. Obes Rev.

[36] Bandy LK, Hollowell S, Harrington R, Scarborough P, Jebb S, Rayner M (2021). Assessing the healthiness of UK food companies’ product portfolios using food sales and nutrient composition data. Scott JA, editor. PLoS One.

[37] Vergeer L, Vanderlee L, Ahmed M, Franco-Arellano B, Mulligan C, Dickinson K (2020). A comparison of the nutritional quality of products offered by the top packaged food and beverage companies in Canada. BMC Public Health.

[38] Douglas N, Knai C, Petticrew M, Eastmure E, Durand MA, Mays N (2018). How the food, beverage and alcohol industries presented the public health responsibility deal in UK print and online media reports. Crit Public Health.

[39] Knai C, Petticrew M, Durand M, Eastmure E, James L, Mehrotra A (2015). Has a public–private partnership resulted in action on healthier diets in England? an analysis of the public health responsibility deal food pledges. Food Policy.

[40] Panjwani C, Caraher M (2014). The public health responsibility deal: brokering a deal for public health, but on whose terms?. Health Policy.

[41] Mindell JS, Reynolds L, Cohen DL, McKee M (2012). All in this together: the corporate capture of public health. BMJ.

