# Correction: Benchmarking the nutrition-related commitments and practices of major Belgian food companies

**DOI:** 10.1186/s12966-022-01304-1

**Published:** 2022-07-29

**Authors:** Iris Van Dam, Naomi Reimes, Stefanie Vandevijvere

**Affiliations:** 1 Sciensano, Service of Lifestyle and Chronic Diseases, Brussels, Belgium; 2 Université Paris-Saclay, INRAE, UR ALISS, 94205 Ivry-sur-Seine, France

**Correction: Int J Behav Nutr Phys Act 19, 43 (2022)**

**https://doi.org/10.1186/s12966-022-01269-1**

Following publication of the original article [1], the authors provided changes in the text and modified the layout of the tables.

The updated text is given below and the changes have been highlighted in **bold typeface**.

The original article [1] has been corrected.

Below is a table of corrections which have been implemented in the original article.

**Table:** 

Section	Originally published text	Corrected text
Abstract - Methods	Commitments were scored according to the BIA-Obesity. To assess company practices, the following indicators were calculated: median Nutri-Score of product portfolios, the proportion of products not-permitted to be marketed to children (using the World Health Organisation Regional Office for Europe nutrient profile model), the proportion of ultra-processed food products (using the NOVA-classification) and the proportion of products displaying Nutri-Score on the front-of-pack. Promotions in supermarket flyers were analysed over a one-year period and quick-service restaurant density around schools was calculated.	Commitments were scored according to the BIA-Obesity. To assess company practices, the following indicators were calculated: median Nutri-Score of product portfolios, the proportion of products not-permitted to be marketed to children (using the World Health Organisation Regional Office for Europe nutrient profile model), the proportion of ultra-processed food products (using the **NOVA classification**) and the proportion of products displaying Nutri-Score on the front-of-pack. Promotions in supermarket flyers were analysed over a one-year period and quick-service restaurant density around schools was calculated.
Abstract – Results	The median proportion of products not-permitted to be marketed to children was 81% (range=12%-100%) and the median proportion of ultra-processed foods was 75% (range=2%-100%) across product portfolios.	The median proportion of products not-permitted to be marketed to children was 81% (range=12%-100%) and the median proportion of **ultra processed** foods was 75% (range=2%-100%) across product portfolios.
Introduction	To date the transparency, comprehensiveness and specificity of the nutrition-related commitments made by the Belgian food industry, both made by individual companies as through overarching industry pledges, have not yet been evaluated. This study set out to benchmark and quantitatively assess the nutrition-related commitments concerning product formulation, labelling, promotion and accessibility made by the biggest Belgian food and non-alcoholic beverage manufacturers, supermarkets and quick-service restaurants, as well as their practices within these same policy domains.	To date the transparency, comprehensiveness and specificity of the nutrition-related commitments made by the Belgian food industry, **both by** individual companies as through overarching industry pledges, have not yet been evaluated. This study set out to benchmark and quantitatively assess the **nutrition related** commitments concerning product formulation, labelling, promotion and accessibility made by the biggest Belgian food and non-alcoholic beverage manufacturers, supermarkets and quick-service restaurants, as well as their practices within these same policy domains.
Methodology	To assess food industry commitments and practices the ‘Business Impact Assessment on Obesity and population-level nutrition’ (BIA-Obesity) was applied. The’Product and brand promotion’ domain considers commitments for reducing the exposure of children to unhealthy food marketing, including the in-store environment of supermarkets and quick-service restaurants. Median scores (range), overall and per BIA-Obesity domain, were calculated including all food industries and separately for food and non-alcoholic beverage manufacturers, supermarkets and quick service restaurants. Practices were assed across the BIA-Obesity domains ‘Product formulation’ and’Product and brand promotion’ for all food industries. Consequently, this company was not discussed within the performance results, but data were included in graphs and tables.	To assess food industry commitments and practices the ‘Business Impact Assessment on Obesity and population-level nutrition’ **(BIA-Obesity) tool and process was** applied. **The ’Product** and brand promotion’ domain considers commitments for reducing the exposure of children to unhealthy food marketing, including the in-store environment of supermarkets and quick-service restaurants. Median scores (range), overall and per BIA-Obesity domain, were calculated including all food industries and separately for food and non-alcoholic beverage manufacturers, supermarkets and **quick-service** restaurants. Practices were assed across the BIA-Obesity domains ‘Product formulation’ **and ’Product **and brand promotion’ for all food industries. Consequently, this company **is** not discussed within the performance results, but data were included in graphs and tables.
Results	The median overall score was 45% (range=14–75%) for food and beverage manufacturers, 46% (range=29–60%) for supermarkets and 15% (range=2–35%) for quick service restaurants (Fig. 1). Products promoted on the cover of the flyers tended to be healthier than the promotions throughout the entire flyers. Data are presented in Table 3.	The median overall score was 45% (range=14–75%) for food and beverage manufacturers, 46% (range=29–60%) for supermarkets and 15% (range=2–35%) for **quick-service **restaurants (Fig. 1). Products promoted on the cover **of flyers** tended to be healthier than the promotions throughout the entire flyers. Data are presented in Table 3.

## References

[1] Van Dam I (2022). Benchmarking the nutrition-related commitments and practices of major Belgian food companies. Int J Behav Nutr Phys Act..

