# Supplementary material for: Benchmarking the nutrition-related commitments and practices of major Belgian food companies
Source: Int J Behav Nutr Phys Act. 2022 Apr 7;19:43. doi: 10.1186/s12966-022-01269-1 (PMC8991492; doi:10.1186/s12966-022-01269-1)
Supplement: Supplementary file 2 — Additional file 2: Supplementary file 2. Weighting per ‘Business Impact Assessment on Obesity and Population Nutrition’ (BIA-Obesity) domain and food industry (Belgium, 2020). [file 12966_2022_1269_MOESM2_ESM.docx]

**Supplementary file 2:** Weighting per ‘Business Impact Assessment on Obesity and Population Nutrition’ (BIA-Obesity) domain and food industry (Belgium, 2020).

| **BIA-Obesity domains** | **Packaged food and Soft drinks** | **Chain restaurants** | **Supermarkets** |
| --- | --- | --- | --- |
| **Corporate strategy** | 10 | 10 | 10 |
| **Product formulation** | 30 | 25 | 25 |
| **Nutrition labelling** | 20 | 15 | 15 |
| **Product and brand promotion** | 30 | 25 | 25 |
| **Product accessibility** | 5 | 20 | 20 |
| **Relationships with other organisations** | 5 | 5 | 5 |
| **TOTAL** | **100** | **100** | **100** |
