# Supplementary material for: Benchmarking the nutrition-related commitments and practices of major Belgian food companies
Source: Int J Behav Nutr Phys Act. 2022 Apr 7;19:43. doi: 10.1186/s12966-022-01269-1 (PMC8991492; doi:10.1186/s12966-022-01269-1)
Supplement: Supplementary file 3 — Additional file 3: Supplementary file 3. The 17 food categories included in the World Health Organisation Regional Office for Europe nutrient profile model (WHO-model) [20]. [file 12966_2022_1269_MOESM3_ESM.docx]

**Supplementary file 3:** The 17 food categories included in the World Health Organisation Regional Office for Europe nutrient profile model (WHO-model) (20).

| **Group** | **Name** |
| --- | --- |
| **1** | Chocolate and sugar confectionery, energy bars, and sweet toppings and desserts |
| **2** | Cakes, sweet biscuits and pastries; other sweet bakery wares, and dry mixes for making such |
| **3** | Savoury snacks |
| **4** | Beverages |
| **4A** | a) Juices |
| **4B** | b) Milk drinks |
| **4C** | c) Energy drinks (often contain o.a. guarana, taurine, glucuronolactone and vitamins) |
| **4D** | d) Other beverages (Soft drinks, sweetend beverages) |
| **5** | Edible ices |
| **6** | Breakfast cereals |
| **7** | Yoghurts, sour milk, cream and other similar foods |
| **8** | Cheese |
| **9** | Ready-made and convenience foods and composite dishes |
| **10** | Butter and other fats and oils |
| **11** | Bread, bread products and crisp breads |
| **12** | Fresh or dried pasta, rice and grains |
| **13** | Fresh and frozen meat, poultry, fish and similar +eggs |
| **14** | Processed meat, poultry, fish and similar |
| **15** | Fresh and frozen fruit, vegetables and legumes |
| **16** | Processed fruit, vegetables and legumes |
| **17** | Sauces, dips and dressings |
