# Supplementary material for: Benchmarking the nutrition-related commitments and practices of major Belgian food companies
Source: Int J Behav Nutr Phys Act. 2022 Apr 7;19:43. doi: 10.1186/s12966-022-01269-1 (PMC8991492; doi:10.1186/s12966-022-01269-1)
Supplement: Supplementary file 4 — Additional file 4: Supplementary file 4. Overall median ‘Business Impact assessment on Obesity and Population Nutrition’ (BIA-Obesity) scores across countries where data were collected for food and beverage manufacturers, supermarkets and quick-service restaurants and companies had the opportunity to verify and complete the publicly available data [11, 12, 28]. [file 12966_2022_1269_MOESM4_ESM.docx]

**Supplementary file 4:** Overall median ‘Business Impact assessment on Obesity and Population Nutrition’ (BIA-Obesity) scores across countries where data were collected for food and beverage manufacturers, supermarkets and quick-service restaurants and companies had the opportunity to verify and complete the publicly available data (11,12,28).

| Country | Total Score | Corporate strategy | Product formulation | Nutrition labelling | Product and brand promotion | Product accessibility | Relationships with other organisations | Response rate |
| --- | --- | --- | --- | --- | --- | --- | --- | --- |
| Australia | 41 | 55 | 40 | 54 | 36 | 5 | 44 | 47 % |
| New Zealand | 38 | 55 | 34 | 47 | 35 | 0 | 38 | 48 % |
| Belgium | 35 | 57 | 37 | 32 | 36 | 8 | 33 | 56 % |
| Malaysia | 11 | 28 | 8 | 15 | 0 | 4 | 25 | 18% |
